# 

**Published:** 1859-11

**Authors:** 


### LIST OF COLLABORATORS.

S. G. Armor, M.D., late Professor of Pathology and Clinical Medicine, Missouri Med. College.
John L. Atlee, M.D., late President Pennsylvania State Medical Society.
Walter F. Atlee, M.D., Philadelphia.
Robert G. Barclay, M.D., Jerusalem, Syria.
John Bell, M.D., Philadelphia, formerly Professor of Medicine, Medical College of Ohio.
T. S. Bell, M.D., Professor of Medicine, University of Louisville.
S. M. Bemiss, M.D., Professor of Clinical Medicine, University of Louisville.
L. P. Blackburn, M.D., New Orleans, La.
George C. Blackie, M.D., Professor of Botany, University of Nashville.
G. C. Blackman, M.D., Professor of Surgery, Medical College of Ohio.
J. L. Bobbs, M.D., formerly Professor of Anatomy, Indianapolis.
W. S. Bowen, M.D., New York.
N. Bozeman, M.D., Montgomery, Ala.
J. T. Bradford, M.D., Augusta, Ky.
John H. Brinton, M.D., Lecturer on Operative Surgery, Philadelphia.
T. M. Carnochan, M.D., Professor of Surgery in the New York Medical College.
W. S. Chipley, M.D., Professor of Medicine, Transylvania University.
A. Clapp, M.D., New Albany, Ia.
D. B. Cliffe, M.D., Franklin, Tenn.
J. Da Costa, M.D., Lecturer on the Practice of Medicine at the Philad. Med. Association.
Samuel H. Dickson, M.D., Professor of Medicine, Jefferson Medical College.
M. Dupierris, M.D., Havana, Cuba.
Richard J. Dunglison, M.D., Philadelphia.
W. F. Edgar, M.D., United States Army.
S. C. Farrar, M.D., Jackson, Miss.
C. S. Fenner, M.D., Memphis, Tenn.
Emil Fischer, M.D., Philadelphia.
Austin Flint, M.D., Professor of Clinical Medicine, Auscultation and Percussion, New Orleans School of Medicine.
Charles Frick, M.D., Baltimore.
W. Y. Gadbury, M.D., Benton, Miss.
O. C. Gibbs, M.D., Chautauque County, New York.
Dr. J. P. Hall, Glasgow, Ky.

WILLIAM A. HAMMOND, M.D., United States Army.

JOHN HARDIN, M.D., Professor of Obstetrics, Kentucky School of Medicine, Louisville.

EDWARD HARTSHORNE, M.D., One of the Surgeons to Wills Hospital for Diseases of the Eye, Philadelphia.

E. B. HASKINS, M.D., Professor of Chemistry, Shelby Medical College, Nashville, Tenn.

C. A. HENTZ, M.D., Marianna, Florida.

ADDINELL HEWSON, M.D., One of the Surgeons to Wills Hospital for Diseases of the Eye, Philadelphia.

SAMUEL L. HOLLINGSWORTH, M.D., late Editor of the Medical Examiner, Philadelphia.

WILSON JEWELL, M.D., Philadelphia, President of the Quarantine and Sanitary Convention.

WM. V. KEATING, M.D., Lecturer on Obstetrics and the Diseases of Women, at the Philadelphia Medical Association.

JOHN G. KERR, M.D., Canton, China.

G. A. KUNKLER, M.D., Madison, Ia.

BEN. LOGAN, M.D., Shreveport, La.

A. LOPEZ, M.D., Mobile, Ala., formerly President of the Alabama State Medical Society.

J. AITKEN MEIGS, M.D., Professor of the Institutes of Medicine in the Medical Department of Pennsylvania College.

S. WEIR MITCHELL, M.D., Lecturer on Physiology at the Philad. Med. Association.

GEORGE R. MOREHOUSE, M.D., Philadelphia.

B. I. RAPHAEL, M.D., New York.

J. C. REEVE, M.D., Dayton, Ohio.

L. C. RIVES, M.D., formerly Professor of Midwifery, Medical College of Ohio.

J. LAWRENCE SMITH, M.D., Professor of Chemistry, University of Louisville.

W. C. SNEED, M.D., Frankfort, Ky., late President Kentucky State Medical Society.

C. H. SPILMAN, M.D., Harrodsburg, Ky., late President of Kentucky State Medical Society.

GEO. SUTTON, M.D., Aurora, Ia.

W. L. SUTTON, M.D., Georgetown, Ky., formerly President Kentucky State Medical Society.

HORATIO R. STORER, M.D., formerly one of the Physicians to the Boston Lying-in Hospital, Boston, Mass.

S. THOMPSON, M.D., Albion, Ill., late President of the Illinois State Medical Society.

L. BEECHER TODD, M.D., Lexington, Kentucky.

E. WILLIAMS, M.D., Cincinnati, Ohio.

### Bi-Monthly Bibliographical Record.

AMERICAN.

A Practical Treatise on the Diagnosis, Pathology, and Treatment of Diseases of the Heart. By Austin Flint, M.D., Professor of Clinical Medicine in the New Orleans School of Medicine. 8vo., pp. 473. Philadelphia: Blanchard & Lea, 1859. (From the author and publishers.)

An Introduction to Practical Pharmacy: Designed as a Text-Book for the Student, and as a Guide for the Physician and Pharmaceutist. By Edward Parrish. 2d edition. 8vo., pp. 720. Philadelphia: Blanchard & Lea, 1859. (From the publishers.)

A Practical Treatise on Operative Dentistry. By J. Taft. 8vo., pp. 383. Philadelphia: Lindsay & Blakiston, 1859. (From the publishers.)

Elements of Medical Jurisprudence. By Theodric Romeyn Beck, M.D., LL.D, Professor of Materia Medica in the Albany Medical College, etc.; and John B. Beck, M.D., Professor of Materia Medica and Medical Jurisprudence in the College of Physicians and Surgeons of the City of New York, etc. With Notes by an Association of the friends of Doctors Beck. The whole revised by C. R. Gilman, M.D., Professor of Medical Jurisprudence in the College of Physicians and Surgeons of New York. Eleventh edition. Philadelphia: J. B. Lippincott & Co., 1860. (From the publishers.)

The Physician's Hand-Book of Practice for 1860. By Wm. Elmer, M.D., and Louis Elsberg, M.D. New York: Townsend & Co., 1859. (From the publishers.)

Quarterly Summary of the Transactions of the College of Physicians of Philadelphia. From Sept. 1st, 1858, to Feb. 2d, 1859, inclusive. 8vo., pp. 150. Philadelphia, 1859. (From the Society.)

Seventeenth Annual Report of the Superintendent of Hamilton County Lunatic Society, for the year ending June 5th, 1859. By Wm. Mount, M.D. 8vo., pp. 18. Cincinnati, 1859. (From the author.)

Humboldt's Life and Character. An Address before the Linnæan Association of Pennsylvania College. By Alfred Stillé, M.D. 8vo., pp. 38. Philadelphia, 1859. (From the author.)

Proceedings of the Academy of Natural Sciences of Philadelphia. 8vo., pp. 27. Philadelphia, 1859. (From the Secretary.)

FOREIGN.

A Treatise on Medical Electricity, Theoretical and Practical: and its Use in the Treatment of Paralysis, Neuralgia, and other Diseases. By J. Althaus, M.D. 8vo., pp. 350. London: Trübner & Co., 1859. (From the publishers.)

Pathological and Practical Researches on Diseases of the Alimentary Canal, Œsophagus, Cæcum, and Intestines. By S. O. Habershon, M.D., F.R.C.P., etc. 8vo., pp. 312. Philadelphia: Blanchard & Lea, 1859. (From the publishers.)

The Use and Abuse of Tobacco. By John Lizars. From the Eighth Edinburgh Edition. 12mo., pp. 138. Philadelphia: Lindsay & Blakiston, 1859. (From the publishers.)

Lectures on Surgical Pathology, delivered at the Royal College of Surgeons of England. By James Paget, F.R.S., etc. Second American edition. 8vo., pp. 700. Philadelphia: Blanchard & Lea, 1859. (From the publishers.)

Gustaf Von Düben's Treatise on Microscopical Diagnosis. Translated, with additions, by Louis Bauer, M.D. 8vo., pp. 82. New York: John Wiley, 1859. (From the publisher.)

A System of Dental Surgery. By John Tomes, F.R.S., etc. 8vo., pp. 686. Philadelphia: Lindsay & Blakiston, 1859. (From the publishers.)

Sketch of the Medical Topography, or Climate and Soils of Bengal and the Northwest Provinces. By John McClelland, F.L.S. post 8vo., pp. 148. London: Churchill, 1859.

Notes on the Wounded from the Mutiny in India. By George Williamson, M.D., etc. 8vo., pp. 124. London: Churchill, 1859.

Remarks on the Anatomical Relations between the Mother and the Fœtus. By Henry Madge, M.D. pp. 49. London: Churchill, 1859.

A Manual of Operative Surgery on the Dead Body. By Thomas Smith, F.R.C.S., etc. post 8vo. London: Longman & Co., 1859.

Traité Clinique et Thérapeutique de l'Hystérie. Par P. Briquet. 8vo. Paris: Bossange et Fils, 1859.

### INDEX.

ABDOMEN, fibrous tumor of, 316.
myeloid tumor of, 317.
Abortion, criminal, 64, 260, 446, 643, 833, 1033.
Abscess, retro-pharyngeal, 330.
of anterior mediastinum, 1078.
of bone, 343.
of liver, 330.
Acetate of copper in pruritis, 181.
Acetic acid in scarlet fever, 140.
Acid, hydrochloric, external use of, 180.
nitrate of silver, 908.
Acrania, 324.
Adams, Robert, treatise on rheumatic arthritis by, review of, 239.
Addison, Thos., M.D., treatise on diseases of supra renal capsules by, review of, 193.
Air-passages, treatise on diseases of, by Green, review of, 628.
Albumen, digestion of by pancreas, 908, 1073.
Albuminous anasarca, tannin in, 718.
Albuminuria in yellow fever, 718.
Alison, W. P., M.D., death of, 1133.
Allin, C. M., on retro-pharyngeal abscess, 330.
Aloes enemata in uterine catarrh, 175.
Amenarius, Dr., on propylamin in rheumatism, 180.
American Medical Association, proceedings of, 755.
transactions, review of, 385.
Amnios, newly discovered function of, 533.
Amputation, causes of death after, 545.
in cancer of penis, 1068.
new mode of dressing wounds of, 888.
of shoulder-blade as a method of embryotomy, 1080.
Amyloid corpuscles on the skin, 746.
degeneration of kidneys, 902.
Anæsthesia produced by iodoform, 731.
by electricity, 956.
Anatomy, treatise on, by Wilson, notice of, 63.
Anatomy, by Gray, review of, 254, 1022.
Aneurism, femoral, cured by digital compression, 73.
aortic, 500, 682.
ischiatic, 1066.
popliteal, cured by compression, 549, 747.
popliteal, treated by forced flexion of knee, 711, 893, 922.
subclavian, treated by injection, 304.
Angina, diphtheritic, 739.
Animal parasites, treatise on, by Kuchenmeister, review of, 437.
Antiseptic for anatomical purposes, 190.
Antonelli, M., on antidote to phosphorus, 733.
Anus, treatise on diseases of, by Ashton, review of, 1.
prolapse of, in children, 715.
Aorta, aneurism of, 500, 682.
compression of in uterine hemorrhage, 753.
Apoplexy, cases of, 328, 494.
of cerebellum, 353.
Appendix vermiformis, communication of with ileum, 117.
Aran, Prof., on uterine catarrh, 175.
Armor, S. G., M.D., on melænal discharges from bowels, 862.
Army, medical appointments in, 765.
British, Medical and Surgical History of in Crimean War, review of, 769.
Arnoux, Dr., on albuminuria in yellow fever, 718.
Arterial obstruction a cause of gangrene, 110.
Artery, splenic, ossification of, 105.
Arthritis, rheumatic, treatise on, by Adams, review of, 239.
Arrow-poison, South American, 940.
Asbestos, local application of, 729.
Ashton, T. J., treatise on anus and rectum by, review of, 1.
Assimilation, defective, in infants, 1089.
Asthma, treatment and clinical history of, 1117.

Atelectasis pulmonum, 337.
Atlee, J. L., M.D., removal of ovarian tumor by, 530.
Atlee, W. F., M.D., account of monstrosity by, 509.
Auscultatory phenomenon, newly discovered, 933.
Ayres, D., M.D., operation for extrophy of bladder by, 709.

Bacon, J., M.D., on epithelial nuclei in urine, 339.
Balassa, Prof., on calculous diseases of Hungary, 917.
Bamberger, Prof., on dilatation of bronchi, 894.
Bang, O., M.D., on curability of tubercular meningitis, 1087.
Barensprung, Prof. V., on prurigo, 903.
Barker, W. O., M.D., on dysmenorrhœa, 530.
Barlow, Dr., on iodide of zinc in chorea, 126.
Barnes, R., M.D., on physiology of placenta prævia, 946.
Barton, E. H., M.D., death of, 1134.
Barudel, M., on perchloride of iron in urethritis, 181.
Batchelder, J. P., M.D., on compressed sponge, 882.
Baths, treatise on, by Bell, review of, 615.
Bauer, L., M.D., on softening of intervertebral cartilages, 343.
Bazin, Dr., lectures on parisitic cutaneous affections, review of, 1026.
Beale, L., treatise on microscope by, notice of, 611.
Beau, Prof., on carbonate of lead in phthisis, 897.
Beau, on uterine hemorrhage, 1085.
Behrend, on spina bifida, 1100.
Bell, Jacob, M.D., necrological notice of, 958.
Bell, Jno., M.D., treatise on baths by, review of, 615.
  lecture on medical heroism by, notice of, 953.
Belladonna in croup, 139.
  prevention of scarlet fever by, 144.
Bennett, J. H., M.D., treatise on physiology by, review of, 577.
Berard, M., death of, 383.
Bernard, Cl., treatise on physiology by, review of, 577.
  on functions of placenta, 533.
  on glucogenetic material, 742.
  on physiology of secretion, 743.
Berton, E. M., on syphilis, 359.
Bertulus, Dr., on hepatitis, 902.
Bibron's antidote, 187.
Bigelow, J., M.D., treatise on practical medicine by, notice of, 430.
Bird, G., M.D., treatise on urinary deposits by, review of, 806.
Birkett, F., on injury of spine, 889.
Blackman, G. C., M.D., on exsection of sciatic nerve, 298.
  case of hermaphroditism by, 324.
Black vomit, microscopic examination of, 106, 347.
Bladder, extrophy of, operation for, 709.
  hernial dilatation of, 313.
  cases of rupture of, 338.
  cancer of, 496.
  foreign bodies in, 1101.
Blockley Hospital, clinical instruction in, 189.
  appointments to, 189, 955.
Blood, temperature of in carotids, 741.
  cause of piling of corpuscles of, 741.
  changes produced in by fever, 348.
  crystals, value of in legal medicine, 939.
  history of discovery of circulation of, by Flourens, notice of, 830.
Boeck, Prof., on syphilization, 156.
Boling, W., M.D., necrological notice of, 766.
Bonamy, Dr., on ulceration and perforation of diaphragm, 146.
Bond, Dr. H., death and autopsy of, 696.
Bones, diseases of, 343.
  fœtal, found in peritoneal cavity, 505.
Bonnet, M., death of, 383.
Bosarelli, M., on antidote to phosphorus, 733.
Boston, letter from, by the senior editor, 950.
Bouchut, M., on tubage of larynx in croup, 174.
Bouisson, M., on buccal cancer in smokers, 1069.
Boursier, Dr., on eclampsia, 178.
Bowditch, H. J., M.D., on diaprhagmatic hernia, 337.
Bowels, nature of green discharges from, 862.
Bowen, W. S., M.D., on softening of bone, 343.
Bozzell, J., M.D., on occlusion of vagina, 526.
Brain, meninges of, tubercle of, 1056.
Brainard, D., M.D., on injection of iodine in hydrocephalus, 713.
  in spina bifida, 1070.
Brandes, Dr., on pneumonia, 897.
Brattler, Dr., on conditions of urine in disease, 534.

Braun, on induction of premature labor, 1084.
Breast, recurring cancer of, 684, 700.
anomalous tumor of, 304.
Brewer, J. H., M.D., on ovarian dropsy, 531.
Briand's manual of legal medicine, review of, 962.
Bright, Dr. Richard, necrological notice of, 382.
Bright's Disease, 356.
Brinton, J. H., M.D., on microscopical examination of tumors, 326.
Brinton, Wm., M.D., treatise on diseases of stomach, by, review of, 1000.
on action of pancreatic juice on albumen, 1073.
British Army in the Crimea, treatise on medical history of, review of, 769.
Brokaw, F. V. L., on abscess of liver, 330.
Bronchial casts, expectoration of, 1064.
Bronchial tubes, minute anatomy of, 938.
foreign body in, producing gangrene of lung, 854
dilatation of, 894.
Bronzed skin, various works on, review of, 193.
Brooke, Mr., on removal of tongue by écraseur, 922.
Brown, B., M.D., on diseased vertebræ, 343.
Brown, M. F., M.D., on rupture of uterus, 507.
Brown-Séquard, E., treatise on supra-renal capsules by, review of, 193.
on effects of cold on human body, 352.
Brown, W. T., M.D., on adulterated milk, 521.
Brunel, Mr., death of, 1134.
Brussels, transactions of Ophthalmological Congress of, review of, 22.
Bryant, Thos., statistics of amputation by, 545.
Bryk, Prof, on value of blood crystals in legal medicine, 939.
Buccal cancer in smokers, 1069.
Budd, C. A., M.D., account of large fœtus by, 514.
Budge, J., on increase of muscle during growth, 917.
Burdach, Dr., on facial neuralgia, 730.
Burnett, W. J., M D., on metamorphosis of tissues, 326.
on enchondroma, 326.
on cheloidea, 345.
on Bright's disease, 339.
Burns, treatment of, 542.
Busch, Dr., on digestion, 532.
on powder wounds, 541.
Bushe, Dr., treatise on rectum by, plagiarism from by Ashton, 1.
Byford, W., M.D., on examination of fœtal circulation, 520.
Byrd, H. L., M.D., on effects of morphia, 529.

Cæsarean section, successful case of, 707.
Calcareous deposits in pleura, 684.
Calculous diseases in Hungary, 917.
Cancer, statistics of, 551.
buccal, in smokers, 1069.
microscopic characters of, 326.
of abdominal viscera, 330.
of bladder, 496.
of hand, 495.
of lip, 749.
of liver and supra-renal capsules, 102, 104.
of mammary gland, 684, 700.
of omentum, stomach, etc., 100.
of penis, 1068.
of pylorus, 106.
of rectum, 307.
of testicle, excision of, 1090.
of tongue, 163, 165, 922.
of uterus, 498, 527.
Capsules, supra-renal, various treatises upon, review of, 193.
disease of, 336.
Carbonate of lead in phthisis, 897.
Carbonic acid, injection of into uterus, 358.
Cardiac hypertrophy and dilatation, remarks on, 1046.
Cardialgia, observations on, 168.
Carnochan, J. M., M.D., contributions to surgery by, review of, 256.
Carroval, action of, 940.
Cartilages, softening of, 343.
Cartwright, S. A., M.D., on pathology of yellow fever, 348.
Casselberry, Dr., on acetic acid in scarlet fever, 140.
Castration for displacement of testicle, 167.
for epilepsy, 365.
Catalogue of Dr. Mott's museum, notice of, 250.
Catamenia during pregnancy, 525.
Cataract, congenital, operation for, 85.
two modifications of operation for, by Grafe, 887.
Catheterism, uterine, in labor, 1084.
sulphate of zinc in, 1104.
Caylus, Prof., on action of solanin and dulcamara, 731.
Cerebellum, apoplexy of, 353.
Cerebral disease, cases of, 328.
Cerebro-spinal meningitis, 120.

Cerebrum and cerebellum, tubercles in, 91.
Cervix uteri, cancroid ulcer of, 527.
hypertrophy of, 734.
severance of during labor, 1060.
inflammation of, treatment of, 1085.
Chapman, E. N., M.D., on puerperal convulsions, 528.
Cheloidea, case of, 345.
Chemistry, treatise on, by Fownes, notice of, 831.
Chenevier, Dr., on rheumatism of diaphragm, 356.
Chest, management of shoulders in examination of, 752.
Chest, wound of, with protrusion of stomach into, 879.
Children, report on diseases of, by Condie, 120.
remittent fever of, 134.
treatise on diseases of, by Condie, notice of, 446.
China, new poison from, 942.
China, state of medicine in, by Kerr, 282.
Chlorosis, new theory and treatment of, 733.
Cholera, Asiatic, pathology of, 346.
Cholera infantum, 127, 130, 131, 132.
Chorea, iodide of zinc in, 126.
cauterization in, 1079.
observations on, by Skoda, 535.
sulphate of zinc in, 1104.
tartar emetic in, 179.
Christison, Robt., M.D., account of a new poison by, 942.
Cimicifuga racemosa, new preparation of, 189.
Circulation, fœtal, mode of examining, 520.
Circulation of blood, history of, by Flourens, translated by Reeve, notice of, 830.
Circulatory apparatus, pathological anatomy of, 333.
Clark, Alonzo, M.D., on black vomit, 348.
on remittent fever, 348.
Clark, Mr. S., on selenite as a substitute for quinine, 907.
Clavicle, intra-uterine fracture of, 686.
Clemens, Dr., on electricity in hernia, 369.
Clinical reports, 1133.
Cock, T. F., M.D., on cerebral disease, 328.
Cod-liver oil, substitute for, 906.
Coffee, diuretic properties of, 538.
Colchicum, in gout, 561.
Cold, effects of, upon human body, 352.
College of Physicians of Philadelphia, donation to, 379.
Collodion, substitute for, 536.
in spina bifida, 1100.
Colon, tubercle of, 685.
Coloring the lips after cheiloplasty, 919.
Compression in treatment of inflammation, 541, 549.
digital, in treatment of aneurism, 73.
in treatment of aneurism, 549.
Condie, D. F., M.D., report on diseases of children, 120.
treatise on diseases of children, notice of, 446.
Convulsions, puerperal, 511, 526, 528.
Copper, acetate of, in pruritis, 181.
Corson, J. W., M.D., on mode of examining chest, 751.
Corvisart, L., essay on function of pancreas, notice of, 636.
essay on function of pancreas, discussion upon, 909.
Costes, Dr., on emphysematous tumors of temporal region, 893.
Craig, Dr., on cerebro-spinal meningitis, 125.
Cramer, Dr., on the preparation of moxas, 364.
Cranium, irregular development of, 324.
irregular development of, in idiots, 328.
Creatine in urine, 339.
Cretenism, 328.
Criminal abortion, paper on, by Storer, 64, 260, 446, 643, 833, 1033.
Crocq, M., on acid nitrate of silver, 908.
Crooks, J. W., M.D., on use of tartar emetic, 526.
Croton oil suppositories in puerperal convulsions, 945.
Croup, tracheotomy in, 136.
glycerin in, 138.
belladonna and mercurial ointment in, 139.
treatment of, 170.
tubage of larynx in, 174, 360.
Crowing inspiration of infants, 230.
Currey, R. M , M.D., case of placenta prævia by, 513.
Cysto-carcinoma, minute anatomy of, 326.

Da Costa, J., M.D., report on pathological anatomy by, 326.
on cancer of pancreas by, 330.
on epithelial tumors, 326.
on anatomy of pneumonia, 337.
on effects of respiration on position of heart, 934.
Dalton, J. C., M.D., treatise on physiology by, review of, 577.
on phosphate of lime in urine, 339.
on structure of placenta, 514.

Davey, J. G., M.D., treatise on nervous system by, review of, 419.
Davis, J. H., M.D., treatise on difficult parturition by, review of, 821.
Davis, N. S., M.D., on blood in fevers, 348.
De Bauvais, M., on Bright's disease, 356.
De Brauw, Dr. J., on ligation of extremities in intermittent fever, 370.
Delirium tremens, meningitis in, 313.
Dermoid cysts, 542.
Diaphragm, ulceration and perforation of in peritonitis, 146.
rheumatism of, 356.
Diaphragmatic hernia, 337.
Diarrhœa, infantile, raw meat in, 559.
Dickson, Prof. S. H., introductory lecture by, notice of, 568.
treatise on practical medicine by, notice of, 832.
Digestion of albuminous matters by pancreas, discussion upon, 908.
Digestive apparatus, morbid anatomy of, 330.
Digital compression in inflammation, 541.
in aneurism, 73.
Diphtheria, treatises upon, review of, 409.
saline injections in, 1080.
Diphtheritic angina in children, 739.
Discovery of circulation of blood, history of by Flourens, notice of, 830.
Dislocations, compound, 149.
of vertebræ, 892.
Diuretic properties of coffee, 538.
Dixon, James, treatise on the diseases of the eye, review of, 1032.
Dowler, B., M.D., notes on pathological anatomy by, 333.
Dropsy consecutive to typhoid fever, 716.
Dropsy, ovarian, cases cured, 531.
Drowning, treatment of, 191.
Dugas, L. A., M.D., on ischiatic aneurism, 1066.
Dulcamara, action of, 731.
Dumb-bell urinary crystals, 339.
Durgin, J. F., M.D., on catamenia during pregnancy, 525.
Durkee, S., M.D., on sarcina ventriculi, 328.
Durkee, Silas, M.D., treatise on gonorrhœa and syphilis, review of, 1015.
Dysentery a cause of hepatic abscess, 330.
Dysmenorrhœa, remedy for, 529, 530.

Ear, blood tumors of pavilion of, 1068.
Eclampsia at the eighth month, 178.
Ecraseur, use of in ovariotomy, 530.
removal of tongue with, 922.
Edibles in Negroland, 372.
Edwards, H. Milne, treatise on physiology by, review of, 577.
Electricity, use of in hernia, 369.
Elemi, poisonous properties of oil of, 906.
Elliott, G. T., M.D., new midwifery forceps by, 522.
Ely, J. W. C., M.D., on fatty degeneration, 326.
Embryotomy by removal of scapula, 1080.
Emphysematous tumors of temples, 893.
Encephaloid of breast, 701.
Enchondroma, anatomy of, 326.
Epilepsy, treatise on, by Radcliffe, review of, 34.
treated by castration, 365.
Epithelial cancer of penis, 1068.
Epithelial tumors, 326.
nuclei in urine, 339.
Ergotin in phthisis, 181.
Ergot in certain diseases of eyes, 362.
Erichsen, J., treatise on surgery by, notice of, 445.
on sacro-iliac disease, 1093.
Erysipelas, identity of with puerperal fever, 527.
Essays by J. K. Mitchell, M.D., notice of, 640.
Excision of cervix uteri, 527.
of knee-joint, causes of failure of, 885.
of testis for malignant disease, 1090.
Exercise, effect of on respiration and pulse, 742.
Expectoration of bronchial casts, 1064.
Exsection of sciatic nerve, 298.
Extra-uterine pregnancy, 319, 502, 504, 1081.
Extrophy of bladder, operation for, 709.
Eye, diseases of, treatise on, by Dixon, review of, 1032.
Eyelids, local sweating of, 1071.
Eyes, use of laudanum in affections of, 538.

Facial neuralgia, certain cure for, 730.
Falcony, M., on preservation of dead bodies, 190.
Fallopian tubes, inflammation of, a cause of peritonitis, 737.
pregnancy, 319, 501, 505.
Fatty degeneration, 326.
of nerve in tetanus, 107.
of liver, 315.
of kidneys, 902.
Fatty heart, diagnosis of, 925.
Fees for professional services, 1125.
Femoral aneurism, 73.
Femur, intra-capsular fracture of, 320.
Fenger, Prof., on cardialgia, 168.

Fenner, C. S., M.D., on fracture of upper jaw, 880.
Fenner, E. D., M.D., on dysmenorrhœa, 529.
Fever, yellow, albuminuria in, 718.
yellow, morbid anatomy of, 348.
continued, changes of blood in, 348.
continued, clinical report on by Flint, 347.
intermittent, ligature of extremities in, 370.
intermittent, state of urine in, 348.
intermittent, treated with ipecac., 905.
puerperal and erysipelas, identity of, 527.
remittent, in children, 134.
remittent, morbid anatomy of, 348.
scarlet, treatise on by Hood, review of, 230.
prophylatic treatment of, 1086.
typhus, 347.
typhus, fatty kidneys in, 1057.
Fibroid tumors of uterus, extirpation of, 714.
Fibrous tumor of abdominal walls, 316.
Fiddes, A., on extirpation of the tongue, 1095.
Findlay, W. S., M.D., on puerperal convulsions, 526.
Fissure of the sternum, 677.
Fistula of gall-bladder, 330.
Fithian, Dr. J., on veratrum viride in scarlet fever, 141.
Flexions of uterus, 736.
Flint, A., M.D., on typhoid fever, 347.
remarks on cardiac hypertrophy and dilatation, 1046.
treatise on diseases of the heart, notice of, 1033.
Flourens, P., treatise on comparative physiology by, review of, 577.
history of circulation of blood by, translated by Reeve, notice of, 830.
Fœtal circulation, mode of examining, 520.
Fœtal mortality lessened by use of forceps, 948.
Fœtal twins, 509.
Fœtus, large size of, 514, 523.
Forceps, midwifery, advantages of frequent use of, 948.
Foreign bodies in urethra and bladder, 1101.
Foreign body in bronchus inducing gangrene of lung, 854.
Forgeaud, V. J., M.D., essay on epidemic diphtheritis of California by, review of, 409.
Forster, Prof., on identity of meconium and vernix caseosa, 915.
Foucher, M., on vaginitis, 1085.
Fountain, E. J., M.D., on foreign body in bronchus, 854.
Foville, A., M.D., on bloody tumors of ear, 1068.
Fracture of clavicle, intra-uterine, 686.
of skull, 687.
of neck of femur, 320.
of upper jaw-bones, 880.
of vertebræ, 889.
Fractures, treatise on, by Malgaigne, review of, 50.
partial of ulna, 1057.
Francis, J. W., M.D., reminiscences of Dr. Mitchill, notice of, 1131.
Fricke, Chas., M.D., on dumb-bell crystals of urine, 339.
Friedleben, A., on physiology of thymus gland, 916.

Gaillard, Prof. P. C., M D., necrological notice of, 381.
Gall-bladder, fistula of, 330.
Gallizioli, Dr., on treatment of ozæna, 181.
Gallois, M., on oxalate of lime in urine, 728.
Ganglionic nervous system, treatise on, by Davey, review of, 419.
Gangrene of spleen, 95.
from arterial obstruction, 110.
idiopathic, of groin, 111.
of lung from presence of foreign body in bronchus, 854.
Garnier, Dr., on tannin in anasarca, 718.
Gastritis, acute, cases of, 330.
Gholson, Dr. R. A., on cholera infantum, 127, 132.
Glottis, tubage of in croup, 360.
Glucogenetic material, 742.
Glycerin in croup, 138.
in chronic laryngitis, 562.
in inflammation of vulva, etc., 562.
Gobrecht, Wm., M.D., Wilson's Anatomy edited by, notice of, 63.
Goddard, P., M.D., invention of needle for metallic sutures by, 957.
Gonorrhœa, treatise on, by Durkee, review of, 1015.
Gonorrhœal rheumatism, 921.
Grafe, Dr. Von, on diseases of lachrymal sac, 361.
modification of cataract operations by, 887.
on local sweating of eyelids, 1071.
on spasms of eyelids, 1067.
Graham, J. N., M.D., on ulceration of cervix uteri, 520.

Grant, J. H , M.D., on placenta prævia, 510.
Gray, H., treatise on anatomy by, review of, 254, 1022.
Green, H., M.D., collection of prescriptions by, 259.
Green, Horace, M.D., treatise on diseases of air-passages by, review of, 659.
Griffith, Dr. Saml., necrological notice of, 958.
Gross, Prof. S. D., M.D., treatise on surgery by, notice of, 832.
 letter from, 951.
Gross, S. W., M.D., on treatment of aneurism by digital compression, 73.
Guersant, M , on treatment of prolapse of anus, 715.
Gunpowder, in toothache, 730.
Günsburg, Dr., on colchicum in gout, 561.
Gunshot wound of neck, 695.
Guntner, on abscess of anterior mediastinum, 1078.
Guor, Dr., on syphilitic nervous diseases, 725.

Hæmoptysis, case of, 1123.
Hallowell, E., M.D., on prevention of phthisis, 328.
Hammond, W. A., M.D., on state of urine in intermittent fever, 348.
 on South American arrow-poison, 940.
Hamon, Dr., on cauterization for chorea, 1079.
Hand, cancer of, 495.
Handy, Dr., on etiology of cholera infantum, 129.
Harder, R. W., M.D., on extra-uterine pregnancy, 504.
Harley, G., M.D., treatise on bronzed skin, etc. by, review of, 193.
Harper, P. H., on more frequent use of forceps in delivery, 948.
Harris, R. P., M.D., on Fallopian pregnancy, 501.
Hart, E., on flexion treatment of popliteal aneurism, 711.
Harvey, oration on, 1130.
Haughton, Rev. S., on natural constants of urine in man, 1071
Hauner, Dr., on diphtheria, 739.
Hearing, physiology of, 913.
Heart, organic disease of, 302, 334, 688, 693, 726.
 fatty, diagnosis of, 925.
 measurements of, 694.
 movement of, in respiration, 934.
 diseases of, treatise on, by Flint, notice of, 1033.
Heart, wounds of, 299.
Hecker, Prof., extra-uterine pregnancy, 1081.
Hematocele, retro-uterine, 539.
Hemeralopia, observations on, 363.
Hemicrania, etiology and treatment of, 536.
Hemorrhage, uterine, 89, 753, 1085.
Henrich, Dr., on spontaneous hydrophobia, 357.
Hepatitis, etiology and diagnosis of, 902.
Hermaphroditism, 324.
Hernia, theory of, 154.
 strangulated, 314.
 inguinal, 320.
 diaphragmatic, 337, 879.
 treatment of, by electricity, 369.
 of stomach into thorax, 879.
Heroism, medical, lecture on, by Bell, notice of, 953.
Hervieux, M., on treatment of syphilis, 367.
Hillairet, Dr. J. B., on apoplexy of cerebellum, 353.
Hillyer, E., on physiology of menstruation, 517.
Hobbs, Dr., on substitute for cod-liver oil, 906.
Hogg, J., treatise on ophthalmoscope by, review of, 60.
Hood, P., treatise on scarlet fever by, review of, 230.
Hooker, E. M. C., on division of the popliteal nerve, 1099.
Hooping-cough, treatment of, 1087.
Horse-chestnut, oil of, in gout and rheumatism, 537.
Hospital, Pennsylvania, resignations and appointments, 378.
 Philadelphia, appointments to, 955.
 City, (Philadelphia,) appointments to, 958.
Hôtel-Dieu, of Paris, history of, 380.
Hour-glass contraction of uterus, 514.
Hulke, Mr., on strabismus from necrosed bone, 555.
Hungary, calculous diseases in, 917.
Hunter, Charles, on hypodermic treatment of diseases, 1110.
Hunter, John, treatise on venereal disease by, review of, 261, 1015.
Hunter, John, re-interment of, 766.
Hutchinson, J., M.D., treatise on bronzed skin, etc. by, review of, 193.
Hydrocele treated with electricity, 712.
 with iron wire, 1067.
Hydrocephalus, injections of iodine in, 713.
Hydrochloric acid, external use of, 180.
Hydrological tour, 563.
Hydrophobia, morbid anatomy of, 346.

Hydrophobia, spontaneous, case of, 357.
Hymen, impregnation without rupture of, 512.
Hypertrophy of pyloric walls, 113.
of cervix uteri, 734.
Hypodermic treatment of diseases, 1110.

Idiots, crania of, 328.
Impregnation without rupture of hymen, 512.
Inflammation, treatment of by digital compression, 541, 549.
of thoracic duct, 898.
Infusoria in secretions from cholera patients, 347.
Intermittent fever, ligature of extremities in, 370.
Intervertebral cartilages, softening of, 342.
Intestine, strangulation of, 1062.
Intestines, cancer of, 100.
hemorrhage from in infants, 133.
transposition of, 681.
wounds of, 301.
Iodide of zinc in chorea, 126.
of potassium in ulcers of leg, 363.
Iodine, injection of in hydrocephalus, 713.
and belladonna in inflamed mammæ, 526.
in spina bifida, 1070.
Iodoform as an anæsthetic, 731.
Iowa, meeting of State Medical Society of, 956.
Ipecac. in obstinate intermittents, 905.
Iris, contraction of, the cause of short-sightedness, 704.
Iron, perchloride of, in urethritis, 181.
perchloride of, injection of aneurism with, 304.
persulphate of, mode of preparing, 571.
Iron wires in the omentum, 118.
Isaacs, C. E.. M.D., on cancroid ulcer of uterus, 527.
Ischias, treatment of, 905.
Ischiatic aneurism, 1066.
Isham, R. N., M.D., on puerperal convulsions, 511.

Jacobi, A., on irregular crania, 324.
Jaquett, F. S., M.D., case of large fœtus by, 528.
Jaw-bones, upper, fracture of, 880.
Jenner, statue of, in Paris, 957.
Jobert, on rhythmical muscular contractions, 740.
Johnston, C., M.D., on extra-uterine pregnancy, 502.
Jones, J., M.D., on malarial fever, 348.
Jones, J. W., M.D., on adhesion of placenta, 511.
Journals, medical, establishment of, 984.
changes in, 984.
Jugular veins, obstruction of, 336.
Jurisprudence, obstetric, 64, 260, 446, 643, 833, 1033.

Kendall, J. V., M.D., on cerebro-spinal meningitis, 120.
Kennedy, H., on diagnosis of fatty heart, 925.
Kerr, J., M.D., on Chinese physic and physicians, 282.
Kidney, tubercle of, 1056.
Kidneys of different size, 499.
fatty, amyloid degeneration of, 902.
fatty, in typhus fever, 1057.
Kirkpatrick, J. N., M.D., on fibrous tumor of uterus, 514.
Kletpinsky, Prof., on external use of hydrochloric acid, 180.
on local effects of asbestos, 729.
Knee-joint, causes of failure in exsection of, 885.
Kneeland, J., M.D., on cancer of abdominal viscera, 330.
on fistula of gall-bladder, 330.
Kuchenmeister, F., M.D., treatise on parasites of human body by, review of, 437.
on substitute for collodion, 536.
Kunkler, J. A., M.D., on post-partum hemorrhage, 89.
Küttner, R., M D., on influence of sex on diseases of children, 1086.

Labor, difficult and complicated, 513, 523.
newly-discovered impediment to, 546.
premature induction of, 1084.
Laceration of perineum, 177.
of the uterus, 1052.
Lachrymal sac, treatment of diseases of, 361.
Laffont, M., on toothache, 730.
Langenbeck, Prof., on extirpation of fibrous tumors of uterus, 714.
La Roche, R., M.D., on black vomit, 347.
Larrey, Baron, notice of, 957.
Laryngitis, diphtheritic, 739.
pseudo-membranous, in an adult, 492.
Larynx, ulceration of, 108.
polypus of, 337.
tubage of, in croup, 360.
Laudanum in affections of eye, 538.
Laugier, M., on new mode of dressing amputation wounds, 888.
Lead, carbonate of, in phthisis, 897.
Lebert, Prof., on organic disease of heart, 726.
on dermoid cysts, 542.

Lectures on diseases of women, by West, review of, 216.
Legal medicine, manual of, by Briand, review of, 962.
Leprosy, ancient and modern, 876.
Letters of advice, 1129.
Leucocythæmia, splenic, 721.
Leudet, Dr., on dropsy, 716.
Lever, Dr. J. C. W., necrological notice of, 383.
Levergood, J., M.D., on puerperal convulsions, 511.
Levy, Prof., on embryotomy, 1080.
Lidell, J., M.D., on acute gastritis, 330.
Ligation of extremities in intermittent fever, 370.
Ligature of œsophagus, 744.
Lime, phosphate of, in urine, 339.
Lip, cancer of, 749.
 coloring of, after cheiloplasty, 919.
Lithotomy, Hindoo, 956.
 different methods of, employed in European hospitals, 920.
Liver, cancer of, 104.
 abscess of, 330.
 condition of, in yellow and remittent fevers, 348.
 direct excitation of, 1073.
 fatty degeneration of, 315.
 steatosis of, 1077.
 wounds of, 698.
Lizars, J., on the use and abuse of tobacco, review of, 1029.
Long and short-sightedness, cause of, 704.
Ludwig, C., on temperature of saliva and blood, 741.
Lungs, collapse of, 109.
 collapse of, in children, 337.
 gangrene of, from foreign body in bronchus, 854.
 distribution of blood-vessels of, 938.
Lussinsky, Dr., on croup, 170.
Luxations, compound, 149.
Luys, M., on amyloid corpuscles, 746.

Malgaigne, treatise on fractures by, translated by Packard, review of, 50.
Mammary gland, cancer of, 684, 701.
Mannkopf, Dr. E., on oil of elemi, 906.
Markoe, T. M., M.D., on abscess of bone, 343.
Martin, Prof. E., on puerperal peritonitis, 737.
Mason, J. K., M.D., report on obstetrics by, 501.
Maunoir, M., case of popliteal aneurism by, 893.
McClelland, W. F., M.D., case of Cæsarean section by, 707.
McSherry, R., M.D., midwifery cases by, 516.
Measles, prophylactic treatment of sequelæ of, 1086.
Meconium and vernix caseosa, identity of, 915.
Meddlesome midwifery, 515.
Medical heroism, lecture upon by Bell, notice of, 953.
Medical history of British army in the Crimea, review of, 769.
Medical journals recently established and suspended, 379.
Medical schools, recent appointments in, 378, 569.
Medical students in Europe, 378.
 in United States, 378, 568.
Medicine in China, 282.
 treatise on by Dickson, notice of, 832.
Megrim, observations on, 904.
Meigs, C. D., M.D., treatise on diseases of women by, notice of, 831.
Meigs, J. F., M.D., on atelectasis pulmonum, 337.
Melænal discharges from bowels, 862.
Meningitis, cerebro-spinal, 120.
 in delirium tremens, 313.
 tubercular, 302.
 tubercular, curability of, 1087.
Mercurial ointment in croup, 139.
Merrill, A. P., M.D., on stramonium in dysmenorrhœa, 530.
Merz, Dr., on hemicrania, 536.
Mesenteric glands, abscess of, 117.
 tubercle in, 685.
Metallic sutures, needle for, 958.
Metamorphosis of tissues, 326.
Metzger, Dr., on pneumonia, 355.
Meyer, Dr. J., on rupture of œsophagus, 900.
Michel, M., on microscopical characters of black vomit, 348.
Microscope, treatise on, by Beale, review of, 611.
 utility of, in practical medicine, 470, 658.
Microscopical characters of tumors, 326.
 of urine, 339.
 of black vomit, 348.
Middledorf, Prof., on polypus of œsophagus, 147.
Midwifery cases, 516.
Midwifery forceps, new form of, 522.
Milk, adulterations of, 521.
Miller, W. H., M.D., on uterine disease, 518.
Miltenberger, G. W., M.D., on creatine in urine, 339.
Mineral Springs of South and West, treatise on, by Moorman, review of, 615.

Minot, F., M.D., cases of uterine disease by, 516.
Mitchell, Prof. J. K., essays by, notice of, 640.
Mitchell, S. W., M.D., on uric acid, 339.
on arrow-poison, 940.
Mitchill, S. L., M.D , reminiscences of, notice of, 1131.
Mitral valve, disease of, 688.
Mole, uterine, 523.
Molluscum produced by injury, 345.
Momm, Dr., on vesico-vaginal fistula, 540.
Monro, Dr. A., necrological notice of, 575.
Monsel's salt, mode of preparing, 571.
Monstrosities, 324, 508, 509.
Montanier, Dr. H., on the tampon, 538.
Moore, C. H., on popliteal aneurism, 922.
Moore, W. P., M.D., on obstetric medicine, 523.
Moorman, J. J., M D., treatise on Mineral Springs, review of, 615.
Morland, W. W., M.D., treatise on urinary organs by, review of, 55.
Morris, J. C., M.D., on belladonna, 144.
on creatine in urine, 339.
Morrison, Dr. E. A., on scarlet fever, 139.
Mott, Dr. V., catalogue of museum of, notice of, 250.
Moxas, preparation of, 364.
Muscle, rhythmic contraction of, 740.
increase of, during growth, 917.
Mütter, Prof. T. D., donation to College of Physicians by, 379.
necrological notice of, 571.
Myeloid tumors of abdomen, 317.

Nasca, Prof., on laudanum in ophthalmia, 538.
Navy medical appointments, 765.
Neck, gunshot wound of, 695.
Needle, new form of, for metallic sutures, 957.
Negroland, edibles in, 373.
Nelson, Robt., M.D., case of ovariotomy by, 531.
Nerve, popliteal division of for neuralgia, 1099.
Nerves, fatty degeneration of, in tetanus, 107.
mutual influences of, 743.
Nervous diseases of syphilitic origin, 725.
Nervous system, ganglionic, treatise on, by Davey, review of, 419.
Nervous vertigo, 722.
Netter, Dr., on hemeralopia, 363.
Neuralgia, facial, cure for, 730.
Newton, Dr. G. M., necrological notice of, 575.
Nickles, Dr. M. J., on formation of vivianite in animal organisms, 353.
Nipples, sore, ointment for, 537.
Nitrate of silver, discoloration of skin by, 365.
acid, advantages of, 908.
Nœggerath, E., M.D., on pessaries, 517.
North, N. L., M.D., case of monster by, 508.

Obstetric jurisprudence, contributions to, 64, 260, 446, 643, 833, 1033.
Obstetrics, report on, for 1858, by Mason, 561.
Occlusion of prepuce, 312.
of vagina, 521, 526.
Œdema of glottis, 108.
Œsophagus, polypus of, 147.
ligature of, 744.
rupture of, 900.
Omentum, cancer of, 100.
iron wire in, 117.
hypertrophy of, 314.
Ophthalmological Congress, proceedings of, review of, 22.
Ophthalmoscope, treatise on, by Hogg, review of, 60.
Oppolzer, Prof., on uræmia, 719.
on splenic leucocythæmia, 721.
on diseases of pancreas, 727.
Oration on Harvey, 1130.
O'Reilly, J., M.D., on identity of erysipelas and puerperal fever, 527.
Osborne, J., M.D., on action of muscles, 1072.
Osseous tissue, softening of, 343.
Ossification of splenic artery, 105.
Otorrhœa, 142.
Ovarian dropsy successfully treated, 531.
Ovarian tumor, removal of, with écraseur, 530.
bone in, 523.
Ovaries, morbid anatomy of, 342.
Ovariotomy, cases of, 531.
in Germany, 556.
Overton, Mr., on puerperal convulsions, 945.
Oxalate of lime in urine, 728.
Oxaluria, creatine in, 339.
Ozæna, nitrate of silver in, 181.

Packard, J. H., M.D., translation of Malgaigne on Fractures, by, review of, 50.
on present state of microscopical science, 470, 658.
Pancreas, treatise on functions of, by Corvisart, review of, 636.
discussion upon, 909.
diseases of, 726.

Pancreas, cancer of, 330.
Pancreatic juice, action of, on albumen, 908, 1073.
Paradise of Doctors, a fable, by Bigelow, review of, 330.
Paraplegia, 95.
Parasites of human body, treatise on, by Kuchenmeister, review of, 437.
Parasitic cutaneous affections, by Bazin, review of, 1026.
Parker, E., M.D., on cholera infantum, 128.
Parker, W., M.D., case of polypus of larynx by, 337.
Partridge, R., on displacement of the testicle, 167.
Parturition, difficult, treatise on, by Davis, review of, 821.
Pathological anatomy, notes on, by Dowler, 333.
Pathological Society's proceedings, 91, 299, 492, 677, 1056, 1126.
Penis, epithelioma of, 1068.
Pennsylvania Hospital appointments, 569.
Perchloride of iron in urethritis, 181.
    injection of, in aneurism, 304.
Perforation of diaphragm in peritonitis, 146.
Pericarditis, 312, 693.
Perineum, laceration of, 177.
Peroneus brevis, rhythmic contraction of, 740.
Persulphate of iron, preparation of, 571.
Pessaries, abuse of, 512.
    new form of, 517.
Petrequin, M., on treatment of hydrocele, 712.
Pharyngeal abscess, 330.
Phelps, C., M.D., on fractures of vertebræ, 889
Philosophy, natural, treatise on, by Silliman, notice of, 258.
Phosphates, syrup of, in stomatitis materna, 1053.
Phosphorus, antidote to, 733.
Phthisis with paraplegia, 95.
    ergotin in, 181.
    observations on prevention of, 328.
    carbonate of lead in, 897.
Physic, treatise on, by Watson, notice of, 63.
Physiology, treatise on, by Dalton, review of, 577.
    by Bennett, review of, 577.
    by Bernard, review of, 577.
    by Edwards, review of, 577.
    by Flourens, review of, 577.
Physician's pocket day-book, notice of, 446.
Piorry, Prof., on megrim, 904.
Pitha, on foreign bodies of urethra and bladder, 1101.
Placenta prævia, 510, 513, 516, 871, 946.
    anatomy of, 514.
    adhesion of, 511.
    newly-discovered function of, 533.
Pleura, calcareous deposits in, 684.
Plummer, J. T., M.D., on dumb-bell forms in urine, 339.
Pneumonia and pleuritic effusion in enteric fever, 94.
    morbid anatomy of, 337.
    observations on, by Metzger, 355.
    treatment of, 897.
Poison, a new, from China, 942.
Poisons, treatise on, by Taylor, notice of, 831.
Polypus of œsophagus, 147.
    of larynx, 337.
Popliteal aneurism, 711, 747, 893, 922.
Popliteal nerve, division of, for neuralgia, 1099.
Porter, J. G., M.D., on meddlesome midwifery, 515.
Potassa, urate of, in urine, 339.
Potassium, iodide of, in ulcers of leg, 363.
Powder wounds, treatment of, 541.
Pregnancy, Fallopian, 501, 505.
    extra uterine, 502, 504, 1081.
Prescriptions of American physicians, collection of, by Green, notice of, 259.
Price, P. C., on excision of knee-joint, 885.
Priory, Dr. D., necrological notice of, 959.
Prizes at the Académie de Médecine, 379.
Prizes, medical, award of, 954.
Progress of American Surgery in Europe, 377.
Prolapse of anus in children, 715.
Prurigo, treatment of, 903.
Pruritis, acetate of copper in, 181.
Pseudo-membranous laryngitis in an adult, 492.
Pseudo-membranous sore-throat, account of epidemic of, in California, review of, 409.
Puerperal diseases at Berlin, 152.
    peritonitis, 524, 737.
    convulsions, 511, 513, 526, 528, 945.
    fever identical with erysipelas, 527.
Pulse, influence of exercise upon, 742.
    suspension of, at wrist, in forced extension of forearm, 352.
Purple, S. S., M.D., on acrania, 324.
Pylorus, stricture of, 112.
    cancer of, 116.

Quackery in Paris, 189.
Quarantine and sanitary convention, 761.
Quinine in scarlet fever, 139.
    substitute for, 907.

Radcliffe, C. B., M.D., treatise on epilepsy by, review of, 34.
Raw-meat in diarrhœa of children, 559.
Rectum, treatises on diseases of, by Ashton and Bushe, review of, 1.
cancerous stricture of, 307.
scirrhus of, 1061.
Reeve, J. C., M.D., translation of Flouren's history of circulation by, notice of, 830.
on stomatitis materna, 1053.
Respiration, influence of exercise upon, 742.
effect of, upon position of heart, 934.
Retention of urine, 188.
Rheumatic arthritis, treatise on, by Adams, review of, 239.
Rheumatism, propylamin in, 180.
of diaphragm, 356.
oil of horse-chestnut in, 537.
gonorrhœal, 921.
Rhorer, J. S., M.D., on congenital cataract, 85.
Riberi, Prof., on labial cancer, 749.
Robin, C., on red-corpuscles, 741.
Roche, M., on saline injections in diphtheritis, 1080.
Rochester, T., M.D., on tracheotomy, 136.
Rodolfi, Dr., on treatment of hydrocele, 712.
Rokitansky, on steatosis of liver and kidneys, 1076.
Rollet, M., on gonorrhœal rheumatism, 921.
Roser, Prof., on hernia, 154.
Routh, on defective assimilation in infants, 1089.
Russell, Dr. G. W., on vaccination, 145.
Rupture of uterus, 507.
of œsophagus, 900.
Ruta and sabina in uterine hemorrhage, 1085.

Sacro-iliac disease, 1093.
Salter, H., M.D., on asthma, 1117.
Sarcina ventriculi, 323.
Saurel, Dr. C., on ipecac. in intermittent fever, 905.
Scabies, cure for, 730.
Scanzoni, Prof., on injection of carbonic acid into uterus, 358.
Scapula, removal of, 311.
Scarlet fever, 139, 140, 141.
treatise on, by Hood, review of, 230.
Schinzinger, Dr. A., on compound dislocations, 149.
Schmidt, E., M.D., on occlusion of vagina, 521.
Schuh, Prof., on coloring the ps after cheiloplasty, 919.
Sciatic nerve, exsection of, 298.
Scirrhus of rectum, 1061.
Scorbutus, formula for, 1080.
Scoutetten, Prof., on measles and scarlatina, 1086.
Scrotum, tumors of, 342.
Secretion, physiology of, 743.
Selenite a substitute for quinine, 907.
Sex, influence of, on diseases of children, 1086.
Shaw, Alex., on treatment of popliteal aneurism, 711.
Shaw, B. S., M.D., on urate of potassa in urine, 339.
Short-sightedness, physiology of, 704.
Sibley, S. W., statistics of cancer by, 551.
Silliman, B., Jr., treatise on natural philosophy by, review of, 258.
Sim, Robt., M.D., on ancient and modern leprosy, 876.
Simon, Dr. Max, on nervous vertigo, 722.
Simon, G., report on ovariotomy in Germany, by, 556.
Sims, J. M., new uterine elevator by, 519.
Skoda, Prof., on chorea magna, 535.
on scorbutus, 1080.
Skull, fracture of, 687.
Smith, B. T., M.D., complicated case of labor, 513.
Smith, Dr. J. L., on remittent fever in children, 134.
Smith, E., on respiration and pulsation, 742.
Smith, H. H., M.D., case of molluscum by, 345.
Smith, Howard, M.D., on persistence of hymen, 512.
Smith, Lewis, M.D., on hydrophobia, 346.
Smith, S., M.D., on rupture of bladder, 338.
Smokers, buccal cancer in, 1069.
Solanin, therapeutic action of, 731.
Spiegelberg, Dr., on uterine hemorrhage, 753.
on mechanical obstacle to labor, 540.
Spilman, C. H., M.D., case of hernia of stomach by, 879.
Spina bifida, collodion in, 1100.
iodine in, 1070.
Spine, cases of injury of, 889.
Spleen, gangrene of, 95.
rupture of, 336.
Splenic artery, ossification of, 105.
Sponge, compressed, history of uses of, 882.
Springs, thermal, 183.
mineral, of South and West, treatise on, by Moorman, review of, 615.
Squire, T. H., M.D., on cerebro-spinal meningitis, 120.
Stanley, F. A., M.D., on uterine hemorrhage, 514.

Starley, S. F., M.D., case of placenta prævia by, 871.
Steatosis of liver and kidneys, 1076.
Steele, G. G., M.D., case of tubal pregnancy by, 505.
Sternum, congenital fissure of, 677.
Stevens, Dr. N. C., on intestinal hemorrhage, 133.
Stomach, protrusion of, through wound into chest, 879.
Stomatitis materna cured by the syrup of the phosphates, 1053.
Stone, W. H., on treatment of chorea by sulphate of zinc, 1104.
Storer, H. R., M.D., on criminal abortion, 64, 260, 446, 643, 833, 1033.
on cupping interior of uterus, 519.
Strabismus cured by removal of necrosed bone, 555.
Strangulation of intestine, 1062.
Stricture of rectum, 307.
Strictures of urethra, rectum, etc., compressed sponge in, 882.
Stuart, H. M., M.D., on condition of urine in malarial fever, 348.
Supra-renal capsules, cancer of, 102.
case of disease of, 336.
reaction of medullary substance of, 1074.
various treatises upon diseases of, review of, 193.
Surgery, operative, contributions to, by Carnochan, review of, 256.
treatise on, by Erichsen, notice of, 445.
by Gross, notice of, 832.
Surgical anatomy, treatise on, by Gray, review of, 254.
Syme, James, on excision of tongue, 163.
Sydenham Society, 1031.
Syphilis, treatment of, in pregnant women, 359.
treatise on, by Durkee, review of, 1015.
treatment of, 367.
Syphilitic nervous diseases, 725.
Syphilization, 156.

Tampon, use of, 538.
Tannin in albuminous anasarca, 718.
Tartar emetic in chorea, 179.
in obstetrics, 526.
Taste, location of sense of, 531.
Taylor, A. S., M.D., treatise on poisons by, notice of, 831.
Taylor, J. E., M.D., on supra-renal disease, 193, 336.
Teale, T. P., on popliteal aneurism, 747.
Temperature of saliva and blood, 741.
Temporal region, emphysematous tumors of, 893.
Terrill, J. E., M.D., case of monstrosity by, 508.
Terrill, J. E., M.D., case of placenta prævia by, 516.
Testicle, displacement of, 167.
excision of, for malignant disease, 1090.
Tetanus, fatty degeneration of nerve in, 107.
Thermal springs, 183.
Tholozan, M., on effects of cold, 352.
Thoracic duct, inflammation of, 898.
Thorburn, J., M.D., on a peculiar auscultatory phenomenon, 933.
Thudichum, J. L. W., treatise on urine by, review of, 806.
Thymus gland, function of, 916.
Tobacco, use and abuse of, by Lizars, review of, 1029.
Todd, L. B., M.D., on laceration of the uterus, 1052.
Tongue, excision of, 163, 165, 922.
Toothache, remedy for, 730.
Toynbee, J., on transmission of sound through tympanum, 913.
Tracheotomy in croup, 136.
Transactions of American Medical Association, review of, 385.
Transposition of viscera, 681.
Trastour, M., on treatment of ulcers, 363.
Traube, Prof., on fatty amyloid kidneys, 902.
Trochanter minor, fracture of, 320.
Trousseau, M., on ligature of œsophagus, 744.
on retro-uterine hematocele, 539.
on treatment of ischias, 905.
Tubage of larynx, 174.
Tubercles in cerebrum and cerebellum, 91.
in large intestine, 685.
of meninges of brain and kidney, 1056.
Tubercular meningitis, 302.
curability of, 1087.
Tuck, Dr. W. J., necrological notice of, 958.
Tumors of the breast, 304.
bloody, of ear, 1068.
epithelial, 326.
microscopical examination of, 326.
myeloid, of abdomen, 317.
of abdominal walls, 316.
Turnbull, Dr. L., on otorrhœa, 142.
Typhus fever, 347.
fatty kidneys in, 1057.
Tympanum, transmission of sounds through, 913.

Ulcers of leg treated with iodide of potassa, 363.
Ulna, partial fractures of, 1057.
Umbilical cord, curiously twisted, 310.

University of the Pacific, organization of, 570.
Upham, J. B., M.D., on typhus fever, 347.
Uræmia, 719.
Urethra, foreign bodies in, 1101.
Urethritis, perchloride of iron in, 181.
Urinary organs, treatise on by Morland, review of, 55.
Urine, retention of, 188.
condition of, in intermittent and remittent fevers, 348.
inodorous, in Bright's disease, 356.
natural constants of, in man, 1071.
oxalate of lime in, 728.
pathological and microscopical characters of, 339, 534.
treatise on deposits of, by Bird, review of, 806.
treatise on pathology of, by Thudichum, review of, 806.
Uterine catarrh, aloes enemata in, 175.
Uterine disease, cases of, 516, 518.
Uterine elevator, new form of, 519.
Uterine hemorrhage after delivery, 89, 520.
compression of aorta in, 753.
treated by ruta and sabina, 1085.
Uterus, fibrous tumors of, 100, 514, 714.
cancer of, 498.
cupping interior of, 519.
death by injecting carbonic acid into, 358.
laceration of, 1052.
flexions of, 736.
rupture of, 507.
ulceration of, 520, 526, 527.

Vaccination, 145.
Vandeveer, J. H., M.D., on epidemic cholera, 347.
Vagina, occlusion of, 522, 526.
Vaginitis, glycerin in, 562.
treatment of, 1085.
Van Holsbeck, Dr., on sore nipples, 537.
Vanzetti, Prof., on treatment of inflammation by compression, 541, 549.
Veins, morbid anatomy of, 335.
Venereal disease, treatise on, by Hunter, notice of, 260, 1015.
Veratrum viride in scarlet fever, 141.
Vermiform appendage, fistula of, 117.
Verneuil, Dr. A., on suspension of pulse by extension of arm, 352.
Vernix caseasa and meconium, identity of, 915.
Vesico-vaginal fistula, treatment of, 540.
Vertebræ, injuries of, 889.
Virchow, Prof., on puerperal diseases, 152.
on uterine flexions, 936.
Virginia Springs, treatise on, by Moorman, review of, 615.
Vivianite, formation of, in animal organisms, 353.
Vomit, black, microscopical examination of, 106.
Von Maack, Dr., on chlorosis, 733.
Vulpian, on excitation of liver and kidneys, 1073.
on phenomena of detached tails of frogs, 1074.
on reaction of medullary substance of supra-renal capsules, 1074.
Vulva, inflammation of, treated with glycerin, 562.

Wade, W. F., M.D., on diphtheria, 409.
Waring, J. J., on ruptured spleen, 336.
Warlomont, Dr., report of Ophthalmological Congress, review of, 22.
Water-glass, 536.
Waters, A. T. H., on structure of lung, 938.
Watson, Thomas, M.D., treatise on medicine by, notice of, 63.
Weatherly, J. S., M.D., on pessaries, 512.
on iodine and belladonna, 526.
Weisse, Dr. J. F., on raw-meat in infantile diarrhœa, 559.
West, Chas., M.D., on diseases of women, review of, 216.
Whitehead, on hooping-cough, 1087.
Willebrand, Dr. V., on ergot in ophthalmia, 362.
Wilson, E., treatise on anatomy by, notice of, 63.
Windle, J. A., M.D., on puerperal fever, 524.
Winterbottom, Dr. T. M., necrological notice of, 958.
Women, treatise on diseases of, by West, review of, 216.
by Meigs, notice of, 831.
Woodward, J. J., on anatomy of cystocarcinoma, 326.
Worms, Dr. J., on inflammation of thoracic duct, 898.
Wounds of heart, 299.
of amputation, new mode of dressing, 888.
of intestines, 301.
of liver, 698.
of neck, 695.
Wright, C. W., M.D., on long and short-sightedness, 704.

Yeast in scarlet fever, 140.
Yellow fever, morbid anatomy of, 348.
albuminuria with, 718.
Young, D. W., M.D., case of fœtal bones in peritoneal cavity by, 505.
Young, James, M.D., on iron wire in hydrocele, 1067.

Zinc, sulphate of, in chorea, 1104.

